# Hypertrophic Pachymeningitis Possibly Secondary to Otitis Media

**DOI:** 10.1155/carm/4541532

**Published:** 2026-03-04

**Authors:** Bo Wang, Guoyong Qin, Jiafang Wang, Kebin Zeng

**Affiliations:** ^1^ Department of Neurology, The First Affiliated Hospital of Chongqing Medical University, Chongqing, 400016, China, cqmu.edu.cn; ^2^ Department of Neurology, Dazu District People’s Hospital, Chongqing, 402360, China; ^3^ Department of Neurology, Chongqing Hygeia Hospital, Chongqing, 400000, China

**Keywords:** case report, headache, hypertrophic pachymeningitis, immune inflammation, otitis media

## Abstract

This study describes a 46‐year‐old female patient who initially presented with hearing impairment and subsequently developed a headache as the predominant symptom, accompanied by the involvement of multiple cranial nerves, including the optic, vestibulocochlear, and trigeminal nerves. Cerebrospinal fluid pressure progressively increased, and imaging demonstrated progression from unilateral to bilateral involvement of the tentorium cerebelli. Clinically, the patient’s hypertrophic pachymeningitis was considered associated with otitis media. After receiving high‐dose corticosteroid pulse therapy, the patient experienced marked relief of the headache and associated symptoms, which was presumed to be related to postinfectious immune–inflammatory mechanisms.

## 1. Introduction

Hypertrophic pachymeningitis (HCP) is a rare neurological disorder characterized by dural thickening and fibrotic inflammation, leading to a wide range of neurological manifestations [1]. The etiology of HCP is heterogeneous, with reported causes including infections, drug‐induced irritation, malignant infiltration or metastasis, and chronic inflammation related to autoimmune disorders [2–6]. However, not all patients with HCP have an identifiable underlying cause. In some cases, the etiology remains unclear despite imaging, biochemical testing, and cerebrospinal fluid (CSF) analysis, and these are classified as idiopathic HCP [7]. HCP secondary to otitis media is a condition of concern in both neurology and otorhinolaryngology. A 2022 study in otorhinolaryngology identified it as a severe central nervous system complication of otitis media associated with antineutrophil cytoplasmic antibody–associated vasculitis (OMAAV) [8]. Here, we report a case of HCP in a patient who developed a headache following a diagnosis of right‐sided otitis media at another hospital. The disease showed progressive involvement of multiple cranial nerves, with negative antineutrophil cytoplasmic antibody results. This report aims to provide a reference for clinical diagnosis and management.

## 2. Case Report

**FIGURE 2:**
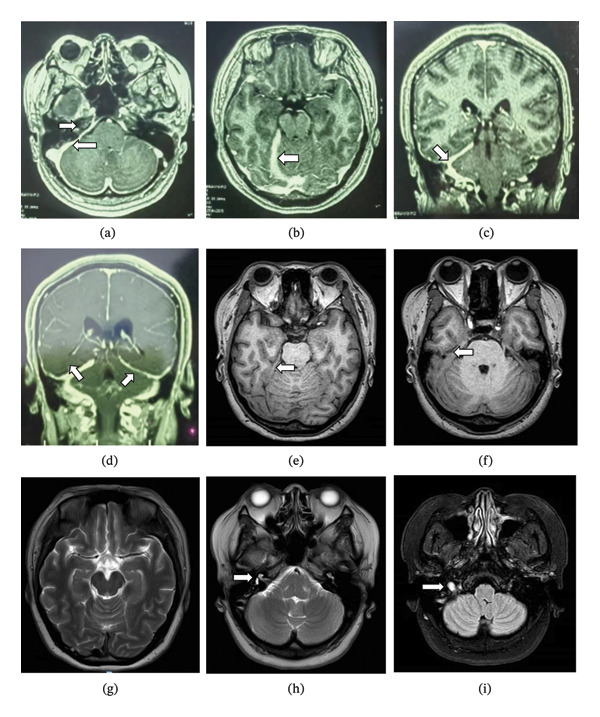
Cranial MRI. Images (a–c) show scans obtained 1 year earlier, and image (d) was acquired 4 months prior. Axial scans (a, b) and coronal scans (c, d) demonstrate marked dural and tentorial thickening in the right posterior cranial fossa, with abnormal signal intensity in the right inner ear. Images (e–i), obtained in August 2025, demonstrate thickening of the right cerebellar tentorium on T1‐weighted imaging (e, f) and abnormal hyperintensity of the right inner ear on T2‐weighted (g, h) and FLAIR (i) sequences.

The patient was a 46‐year‐old woman diagnosed with “otitis media” at a local hospital one year earlier for right‐sided tinnitus and hearing loss. After more than 10 days of antibiotic therapy (details unavailable), she developed progressive right‐sided hearing loss leading to deafness. This was accompanied by intermittent headaches of variable location and nature, occasionally associated with vertigo and vomiting. Physical examination confirmed right‐sided hearing loss (Figure 1); no other abnormal findings were detected. Laboratory tests revealed WBC 11.58 × 10^9/L, neutrophils 75.3%, absolute neutrophil count 8.62 × 10^9/L, and C‐reactive protein 16.73 mg/L. Quantitative immunoglobulins, lupus anticoagulant, rheumatoid factor, antistreptolysin O, antineutrophil cytoplasmic antibodies, and antinuclear antibodies were within normal limits. Serological tests for HIV and syphilis were negative, and serum tumor markers were normal. Lumbar puncture showed a CSF pressure of 220 mmH_2_O, white blood cell count 3.00 × 10^6/L, protein 0.45 g/L, and chloride 131.0 mmol/L. Metagenomic sequencing (genomic and transcriptomic) and testing for central nervous system demyelinating antibodies showed no specific abnormalities. Cranial MRI demonstrated thickening of the tentorium cerebelli and infratentorial meninges, along with right‐sided otomastoiditis, suggesting a possible diagnosis of HCP. Treatment with mannitol and other intracranial pressure–lowering agents alleviated the headache. Eight months earlier, the patient was rehospitalized for recurrent headaches persisting for 1 week. A second lumbar puncture revealed a CSF pressure of 290 mmH_2_O, albumin 0.483 g/L, trace protein 1.13 g/L, and serum IgG 0.458 g/L. High‐dose corticosteroid pulse therapy followed by oral prednisone (60 mg/day) resulted in complete resolution of the headache. After discharge, recurrent headaches were treated at a local hospital with oral prednisone and mannitol, which provided partial relief after each course. Four months earlier, the patient was rehospitalized for episodic occipital pain persisting for over 10 days. Cranial MRI demonstrated diffuse thickening of the bilateral tentorium cerebelli and infratentorial dura (Figure 2).

FIGURE 1Pure‐tone audiometry findings. Panel (a) demonstrates marked impairment of both air and bone conduction in the right ear. Bone conduction was relatively better preserved than air conduction, consistent with mixed hearing loss. Panel (b) demonstrates normal air and bone conduction in the left ear.

**Figure:**
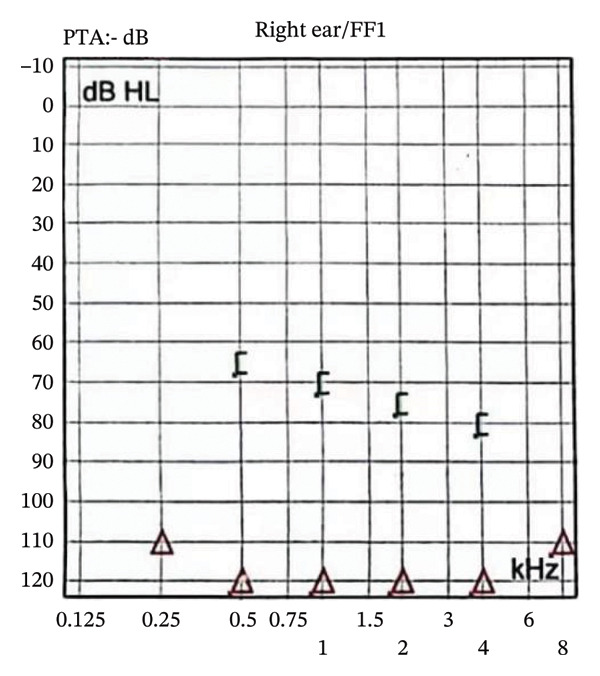
(a)

**Figure:**
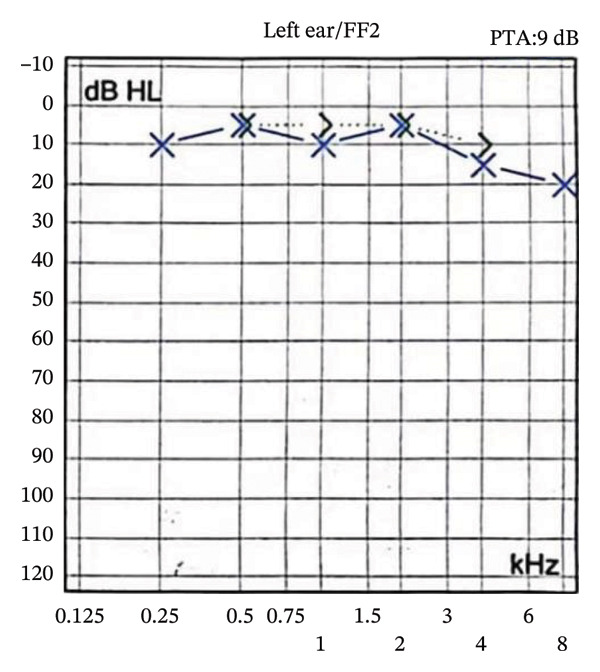
(b)

**FIGURE 3:**
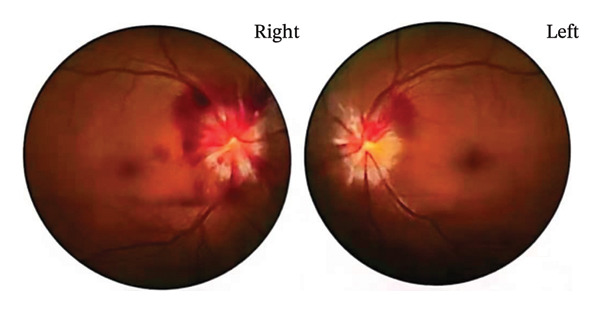
Fundus photographs of both eyes. Bilateral optic disc edema is evident, characterized by blurred margins and peripapillary radial hemorrhages.

In April 2025, the patient was admitted to the neurology department for a 15‐day history of headache and diplopia. She presented with diplopia and right facial hypesthesia at that time. Her medical history was notable for chronic urticaria and prior pulmonary tuberculosis. She was alert, with normal extraocular movements and pupillary light reflexes. Right‐sided hearing loss was present. Limb movements were normal, and both Babinski and meningeal signs were absent. Fundus photography revealed bilateral papilledema with blurred margins and peripapillary radiating hemorrhages (Figure 3). Blood tests were negative for antineutrophil cytoplasmic antibodies, and IgG4 levels were normal. Lumbar puncture showed a CSF opening pressure of 410 mmH_2_O and a protein concentration of 1.18 g/L. On admission, she received high‐dose methylprednisolone (1000 mg/day) for 3 days, followed by gradual tapering. After 1 week, her headache and diplopia markedly improved, and facial sensation normalized; however, hearing remained impaired. She was discharged after 3 weeks of therapy with overall symptomatic improvement.

**FIGURE 4:**
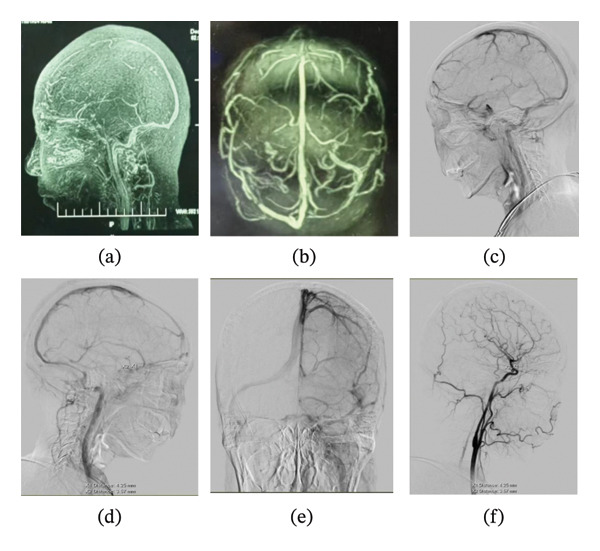
Cerebrovascular imaging. Images (a, b) show MRV scans obtained 4 months earlier. Images (c–f) display digital subtraction angiography (DSA). MRV reveals strip‐like filling defects at the arrow‐marked sites and the absence of straight sinus visualization in the coronal view. Sagittal DSA images (c, d, f) and the coronal image (e) demonstrate absent straight sinus opacification, hypoplasia of the left transverse sinus, and a right paraclinoid internal carotid artery aneurysm measuring about 4.25 × 3.57 mm.

Four months prior, the patient underwent cranial MRV at an external hospital, revealing strip‐like filling defects in the right transverse and sigmoid sinuses and in the right great cerebral vein, suggesting possible thrombosis. During the current admission, cerebral angiography demonstrated left transverse sinus hypoplasia, the absence of straight sinus opacification, and a right paraclinoid internal carotid artery aneurysm measuring approximately 4.25 × 3.57 mm (Figure 4). Cerebral angiography did not reveal the band‐like filling defect previously suggested by cranial MRV. Therefore, the filling defect reported on MRV is most likely an imaging artifact. After her headache and diplopia resolved, the patient was admitted to our neurosurgery department in May 2025 for aneurysm management. Repeat DSA was performed, followed by stent‐assisted endovascular embolization of the right paraclinoid internal carotid artery aneurysm on May 26, 2025. On July 7, 2025, catheter‐based stent‐assisted embolization of another intracranial aneurysm and flow‐diverting device placement for a left internal carotid artery ophthalmic segment aneurysm were performed. During neurosurgical hospitalization, right‐sided hearing loss persisted, whereas other symptoms did not recur. Follow‐up cranial MRI in August 2025 demonstrated dural thickening primarily localized to the right tentorium cerebelli, whereas other dural regions had largely returned to normal. The abnormal hyperintensity in the right inner ear remained unchanged.

## 3. Discussion

Otitis media is widely regarded as a common comorbidity or a potential etiological factor in HCP [9]. Previous studies have demonstrated that chronic otitis media is a frequent clinical manifestation in patients with immune‐mediated or idiopathic HCP [10]. In the present case, the patient had a history of acute otitis media prior to disease onset, with headache persisting throughout the disease course. In the later stages, multiple cranial nerves were affected, including the vestibulocochlear, optic, and trigeminal nerves, and were accompanied by elevated intracranial pressure. Cranial MRI revealed abnormal signal intensity in the right inner ear, while laboratory tests showed an elevated white blood cell count, neutrophil percentage, and C‐reactive protein levels. These findings suggest a close association between hypertrophic cranial pachymeningitis (HCP) and otitis media. However, despite a diagnosis of otitis media and subsequent empirical broad‐spectrum antibiotic therapy at a local hospital, the patient’s clinical symptoms continued to progress. In addition, metagenomic next‐generation sequencing of the CSF revealed no abnormalities. These results indicate a low likelihood of direct infection by pathogenic microorganisms, including bacteria, fungi, or viruses. Therefore, it remains uncertain whether the dural changes are entirely secondary to otitis media, as the available evidence is insufficient. The possibility of idiopathic immune‐mediated HCP coexisting with otitis media cannot be excluded, and a histopathological examination is required for further clarification. However, the patient declined the pathological examination for financial and personal reasons.

In this case, the pathophysiological mechanism of HCP remains unclear. During the current hospitalization, testing for antineutrophil cytoplasmic antibodies was negative, and serum IgG4 levels were within the normal range. However, the patient showed a favorable response to methylprednisolone and prednisone, with complete resolution of meningeal inflammation. This response may be attributed to the potent anti‐inflammatory and immunosuppressive effects of corticosteroids [11]. Taken together, we speculate that the pathogenic mechanism in this patient may involve noninfectious inflammatory responses mediated by undetected or currently unknown inflammatory mediators or immune substrates. Whether this inflammatory response represents a cross‐immune reaction triggered by otitis media requires further confirmation by the pathological examination of the middle ear and meninges.

During this hospitalization, cerebral angiography incidentally revealed an aneurysm in the paraclinoid segment of the right internal carotid artery. Both intracranial aneurysms and HCP may present with headache and papilledema [12, 13]. However, headache associated with intracranial aneurysms is more commonly related to hemorrhage or a local mass effect [14]. Cranial MRI showed no definite hemorrhagic lesions or imaging evidence of subarachnoid hemorrhage. Moreover, intracranial aneurysms do not typically cause papilledema directly. When papilledema occurs, it is usually due to a mass effect from a giant aneurysm or secondary elevation of intracranial pressure after subarachnoid hemorrhage [12, 15]. Specifically, giant intracranial aneurysms are defined as having a maximum diameter greater than 25 mm [16]. Overall, imaging evidence supporting the aneurysm as the cause of the patient’s clinical manifestations is insufficient, and further clinical and radiological follow‐up is required.

This case report has several notable limitations. First, the association between otitis media and HCP was primarily inferred from indirect evidence, including clinical manifestations, cranial MRI findings, and laboratory indicators such as routine blood tests and C‐reactive protein levels. No pathological evidence from dural or brain tissue was available to support this association. Therefore, the coexistence of idiopathic HCP and otitis media cannot be excluded. Second, because of limited resource sharing among medical institutions across regions, detailed information on the patient’s prior treatment and interventions for otitis media at other hospitals was unavailable. In addition, repeat pure‐tone audiometry or related examinations were not performed during subsequent outpatient follow‐up. This limitation precluded a quantitative assessment of changes in the patient’s hearing function. Finally, in the absence of biopsy evidence from the dura mater or brain tissue, the pathogenesis of HCP in this patient could only be inferred from published literature and the treatment response.

In summary, we report a case of HCP in a patient with a history of otitis media preceding disease onset. No specific antibody types were identified in CSF or hematological testing. The patient experienced transient symptomatic improvement after multiple courses of oral prednisone, followed by symptom recurrence. Subsequently, marked clinical improvement was achieved after high‐dose methylprednisolone pulse therapy (1000 mg). No symptom recurrence was observed during neurosurgical follow‐up. These findings suggest that high‐dose corticosteroid pulse therapy may be an effective option when oral prednisone is ineffective. This approach may rapidly shorten the disease course. It may also reduce the risk of complications associated with prolonged immunosuppressive therapy. Additionally, it may help alleviate the patient’s financial burden.

## Author Contributions

Dr. Kebin Zeng and Mr. Bo Wang discussed the research topic and the criteria for case selection. Case collection was primarily managed by Mr. Bo Wang, with Mr. Guoyong Qin and Ms. Jiafang Wang providing supplementary data and refining case and examination records. Mr. Guoyong Qin also contributed to the literature review. The initial manuscript draft was prepared by Mr. Bo Wang, and Dr. Kebin Zeng reviewed it and provided feedback for revision.

## Funding

No funding was received for this research.

## Disclosure

All authors have read and approved the final manuscript.

## Ethics Statement

In this case report, all potentially identifiable patient information was thoroughly deidentified. The study was conducted as a purely retrospective analysis without introducing any additional interventions or procedures involving the patient. All diagnostic and therapeutic measures described were standard treatments administered under the clinical circumstances at that time. The study did not alter the original treatment regimen or impose any additional risks or burdens. Based on these considerations, the Ethics Committee of the First Affiliated Hospital of Chongqing Medical University concluded that this study met the criteria for ethical approval and exemption from informed consent.

## Conflicts of Interest

The authors declare no conflicts of interest.

## Data Availability

Data would be available by contacting the corresponding author.
